# Redeployment of the trainee orthopaedic surgeon during COVID-19: a fish out of water?

**DOI:** 10.1080/17453674.2020.1824155

**Published:** 2020-09-25

**Authors:** Giles Faria, Baha John Tadros, Natalie Holmes, Siddharth Virani, Gaddam Kumar Reddy, Baljinder Singh Dhinsa, Jaikumar Relwani

**Affiliations:** East Kent Hospitals University NHS Foundation Trust, Ashford, Kent, UK

## Abstract

Background and purpose — COVID-19 has had a significant impact on health services and the entire healthcare sector, including trauma and orthopaedics, has been compelled to adapt. At the heart of this was the redeployment of the orthopaedic trainees to support “frontline specialties”. This paper sheds light on the experience of orthopaedic trainees in redeployment.

Methods — In this retrospective study, we asked orthopaedic trainees in the KSS (Kent, Surrey, Sussex) and London Deaneries to complete a survey regarding their experience in redeployment during the COVID-19 outbreak. The study took place in the Kent, Surrey, Sussex, and London regions of the United Kingdom over a period of 8 weeks from 15th of March 2020 until 15th of May 2020. The study was based at East Kent Hospitals University NHS Foundation Trust and participants were recruited from a number of secondary and tertiary care centres across the region. 120 orthopaedic trainees were contacted, working in 21 teaching hospitals. Of these, 40 trainees (30%) from 13 hospitals responded and completed the survey.

Results — 50% of the surveyed trainees were redeployed to other specialties. Trainees spent varying amounts of time in the redeployed speciality and gave differing views on how comfortable they felt and how useful they felt the experience was. One-third of trainees experienced symptoms and/or tested positive for COVID-19 and the majority of these were redeployed to other specialties.

Interpretation — Orthopaedic training appears to have taken a temporary back seat at this time but trainees have made a significant contribution to reinforcing key front-line specialties in the fight against COVID-19.

COVID-19 has had a significant impact on the health services since late December 2019, when the outbreak was reported in Wuhan, China. The entire healthcare sector, including trauma and orthopaedics, has been compelled to adapt to the exponentially increasing number of cases (Chang Liang et al. 2020). In the United Kingdom, the focus of the healthcare system shifted to treating COVID-19 cases and reducing further spread. Hospitals were seeing their resources shifted to reinforce the emergency, acute medical, and intensive care teams as advised by NHS England and the British Orthopaedic Association (2020). This appeared to be consistent with other countries around the world (Sarpong et al. 2020), whose hospitals and orthopaedic services followed a similar tactic (Chang Liang et al. 2020).

At the heart of this was the redeployment of the orthopaedic trainees to support “frontline specialties”. The governing body that manages trainees across all specialties, Health Education England (HEE), issued guidance recommending this redeployment (Health Education England 2020), but this was to be organised locally by trusts.

This had huge repercussions on the workings of the orthopaedic department and the trainees themselves. This paper sheds light on the experience of orthopaedics trainees in redeployment during the COVID-19 outbreak.

## Methods

In this retrospective study, we asked orthopaedic trainees in the KSS (Kent, Surrey, Sussex) and London Deaneries to complete a survey regarding their experience in redeployment during the COVID-19 outbreak. The survey was constructed using Survey Monkey (Palo Alto, CA, USA), and comprised 10 questions (Table 1).

**Table 1.** Outlining survey questions

Redeployment survey
Have you been re-deployed to another specialty during the COVID-19 crisis? What’s your current grade? To which department were you re-deployed? In the last 8 weeks, what percentage of your total clinical time was spent in re-deployment? What did your role involve? How comfortable did you feel performing the role given to you? Did you receive any training/education during this period in that speciality? How valuable did you find your experience in re-deployment? Have you yourself had symptoms and/or tested positive for COVID-19 in the last 8 weeks? Any further comments regarding your experience?

120 trainees were contacted, working in 21 teaching hospitals. Of these, 40 orthopaedic trainees (30%) from 13 hospitals responded and completed the survey. Invitations were sent using email, WhatsApp, and Facebook messenger. A week later a reminder was sent.

As the variables were binary or categorical, data was described using frequencies and percentages.

### Ethics, funding, and potential conflicts of interest

Ethical approval and registration—Not applicable.

Funding sources—No funding sources to declare.

Conflicts of interest—No conflicts of interest to declare.

## Results

**Figure 1.**
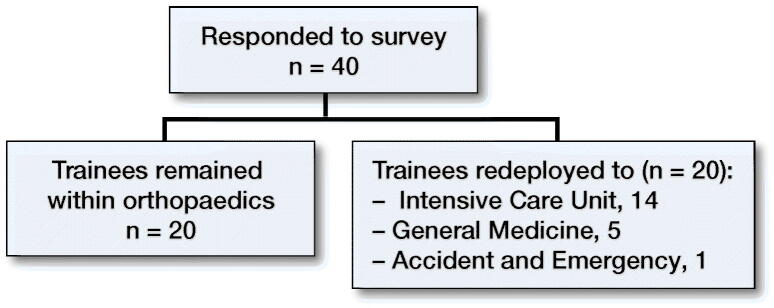
Pattern of redeployment.

Of the 120 trainees surveyed, 40 responses were recorded, and of the 40 trainees who responded, 20 were redeployed (Figure 1).

The time spent in the redeployment role was highly varied. 7 trainees spent a quarter of their time in redeployment, 4 trainees spent half of their time in redeployment, 4 trainees spent three-quarters of their time in redeployment, and 5 spent all their time in redeployment.

On surveying the 20 redeployed trainees’ attitudes on the tasks they were asked to perform, 2 reported that they were “very uncomfortable”, 4 were “fairly comfortable”, 5 were “indifferent”, and 9 were “completely comfortable”.

24 trainees reported having received an induction or training session on COVID-19, with some receiving it prior to their redeployment. The remainder noted an absence of such.

**Figure 2.**
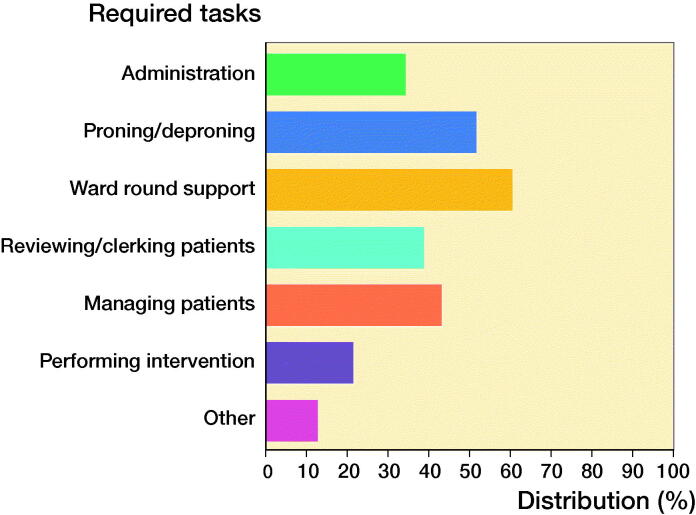
Tasks required of trainees during redeployment.

The typical activities asked of trainees in their redeployment included administrative tasks, proning and de-proning of intubated and ventilated patients, reviewing patients, or assisting with clerking of new patients. Some trainees reported managing patients themselves and performing interventions (Figure 2).

When asked about how useful they found their redeployment with regard to their future careers, 6 trainees found it “interesting but not applicable”, 5 of trainees found it “not interesting and not applicable”, 7 found it “interesting and applicable”, and 2 were unsure.

Working in close proximity to COVID-19 confirmed patients was likely to have been of significant concern to many trainees and one-third of trainees reported experiencing symptoms and/or testing positive for COVID-19 in the last 8 weeks from 15th of March 2020 until 15th of May 2020.

**Figure 3.**
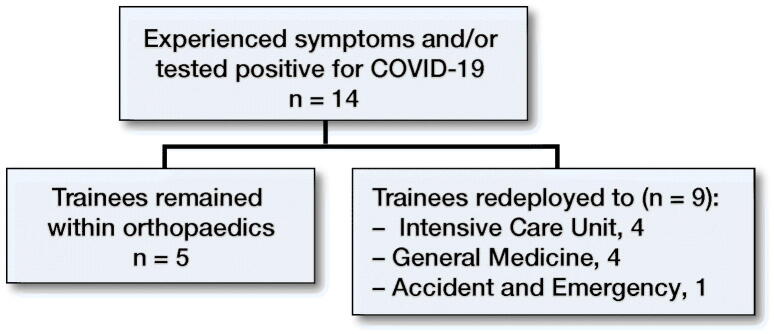
Number of trainees who experienced symptoms and/or tested positive for COVID-19 during redeployment.

Looking into the redeployment status of those who experienced symptoms of, or tested positive for, COVID-19, the majority were redeployed to various other clinical areas such as general medicine or intensive care (Figure 3).

## Discussion

COVID-19 redeployment is commonplace in most hospitals in the United Kingdom. The British Medical Association (BMA) compiled a document outlining how the process should occur (British Medical Association 2020a). In summary, it states that the trainee should consent to the redeployment, understand their role, and not be expected to carry out any activities outside their level of training or that they do not feel comfortable with. Employers are also expected to carry out an induction session specific to the redeployment proposed, and provide adequate personal protective equipment (PPE) in line with Public Health England guidance (Public Health England 2020). This includes a special addendum on what to do if this condition is not met (British Medical Association 2020b).

Our data shows that half of the surveyed trainees were redeployed to other specialties, with the other half remaining in orthopaedics. This was part of a coordinated response of many hospitals to boost the staff and resource allocation to “front line” specialties (Sarpong et al. 2020) and was endorsed by Health Education England (HEE). This appears to be in line with a national survey of all UK doctors performed by the BMA, which reports that half of UK doctors were redeployed to other specialties (British Medical Association 2020c).

Junior trainees were more commonly redeployed and the reasons for this may have included increased retention of general medical knowledge compared with their senior counterparts (Culp and Frisch 2020). It was felt by some university and hospital leaders that the risk of exposure to COVID-19 and the need to conserve PPE as much as possible outweighed the training benefit (Gallagher and Schleyer 2020) to junior surgeons. This was recognised by HEE and the Joint Colleges of Surgical Training (JCST) and the annual review of trainees’ competencies was altered to reflect this. However, this decision did not appear to be favoured by all trainees, with some reporting frustration with the lack of orthopaedic training opportunities:

*“Blanket exclusion from theatre has been frustrating considering the opportunities have continued to exist in some capacity and the redeployment duties have been low-demand.”*

The proportion of the time trainees spent in different specialties was managed locally as per guidance from HEE, which states that trainees may be required to assist in managing acutely unwell patients, at the expense of planned training activites, but with protective expectations about not working beyond their competencies and capabilities (Health Education England 2020). Some institutions redeployed their trainees wholly to front-line specialties such as Accident and Emergency and intensive care, whilst others created a rota with a mix of parent and redeployed specialties. Reasons for the discrepancy may have included varying staffing levels within that hospital and anticipated decline in workload levels within orthopaedics. It is interesting to note that only one-quarter of trainees spent the entire period in redeployment, whilst the majority continued to have some orthopaedic exposure. Therefore, a significant proportion of trainees could at least partially continue their training whilst supporting the response to the pandemic. Reasons for this could be the equitable distribution of the redeployment duties, which would have ensured that the morale of the trainee surgeons was maintained:

*“Short redeployment managed between orthopaedics and ITU. Worth doing, but was glad to return to regular duties.”*

Some trainees felt that they were surplus to requirement in their redeployed specialty. For example, as the number of admissions to intensive care started to decline after the initial wave (ICNARC 2020), workload may have diminished with the added caveat that intensive care trainees may be better placed to carry out tasks and clinical work in their own specialty than orthopaedic trainees:

*“Intensive Care (ITU) was very supportive, though over staffed and little work to do, however the redeployment was understandable. Better use of the resource could have been if surplus staff were sent back to parent specialty for support of those services.”*

1 of the key themes outlined by the BMA in its document surrounding redeployment was to ensure trainees and junior doctors did not perform tasks outside their level of training and competency and were not pressured to do so (British Medical Association 2020a). Our data showed that the majority of trainees were “indifferent” or “completely comfortable” with the tasks they were asked to do. These tasks were mostly completing administrative and documentation aspects of the ward round, assisting with proning and de-proning patients and accompanying intensive care doctors to clerk in new patients. This also suggests a reasonable competency amongst the trainees in carrying out basic medicine-based duties and reflects the value of such doctors in unprecedented situations, such as the pandemic. However, around a quarter of surveyed trainees report they were uncomfortable with their duties and this included being asked to run an A&E minor injuries with no senior support, and managing general medical patients. This appears to be in line with the national BMA survey, which reported that one-fifth of UK doctors were not comfortable with tasks given to them in redeployment (British Medical Association 2020c). Whilst this level of care may be expected of core trainee level doctors, it is understandable that this could be anxiety-provoking (British Medical Association 2020a).

Anxiety amongst doctors was a significant theme in the BMA national survey, which reported that one-third of UK doctors suffered depression, anxiety, stress, burnout, emotional distress, or another mental health condition worse than prior to the pandemic, with 40% having to access NHS well-being services (British Medical Association 2020c). This highlights a key issue surrounding well-being of trainees working during the pandemic.

Another key requirement highlighted by the BMA was to ensure trainees and junior doctors received adequate induction and training sessions to ensure they were as prepared as possible for their redeployment to a completely new specialty (British Medical Association 2020a), possibly one that they have never worked in before. Our results showed that almost half of trainees received no such training, which may have contributed to one-quarter of trainees feeling uncomfortable with the tasks they were asked to perform. The possible reasons for the lack of induction or training could be the rapidly changing requirements of the COVID-19 response, leading to urgent redeployment before delivery of such training was possible:

*“We were provided with little or no training. Departmental ‘Zoom’ teaching was set up once weekly.”*

All trainees surveyed were orthopaedic trainees and therefore it was interesting to ascertain how useful they felt the redeployment would be to their future careers. The majority found the redeployment interesting but half did not feel it was actually applicable to their future careers. Some on the other hand felt supported and gained valuable experience/;

*“Redeployed under the medical team. Great training experience from all grades who were very welcoming—especially given the fact that I was an Orthopaedic trainee.”*

Finally, a likely concern for trainees as outlined by the BMA was the close and prolonged proximity to COVID-19 positive patients and the risk of being exposed to COVID-19 themselves. This was reflected in the BMA national survey, which reported that only four-tenths of UK doctors felt adequately protected from COVID-19 (British Medical Association 2020c). Whilst our survey did not directly address issues around personal protective equipment (PPE), the BMA national survey reported widespread shortages of common items, such as “shortage” or “no supply at all” in 12% of responses. 29% of respondents felt “sometimes” or “often” pressured to see patients without adequate protection for the specific area of care (British Medical Association 2020c).

Our data shows that one-third of trainees surveyed displayed symptoms and/or tested positive for COVID-19 during the survey time and the majority of these trainees were redeployed, with the areas of intensive care and general medicine being the most affected (Figure 3). This appears to be higher than the national survey of UK doctors by the BMA, who reported that roughly one quarter of surveyed respondents displayed symptoms and/or tested positive for COVID-19 (British Medical Association 2020c).

However, the numbers in our study are too small to draw a reliable conclusion. We acknowledge that responder bias may be present in terms of increased number of responses from those trainees who were redeployed compared with those who remained in orthopaedics. Anecdotally, we believe that the low response rate could be due to the large number of COVID-19 related emails that trainees received at this time, as well as altered shift patterns and activities within shifts. Risk factors for this may have included trainees’ health conditions or lack of adequate PPE. However, it is noteworthy that a significant number of trainees who remained within orthopaedics displayed symptoms and/or tested positive for COVID-19 during the survey period and it stands to reason that the risk factors for this are the same regardless of the department.

## Conclusion

Our data provides the first insight into orthopaedic trainees’ experience of redeployment to other specialties in the United Kingdom. COVID-19 has changed the way the entire healthcare system functioned in the 8-week study period from 15th of March 2020 until 15th of May 2020. Whilst orthopaedic training appears to have taken a temporary back seat at this time, trainees have made a significant contribution to reinforcing key front-line specialties, such as intensive care, in the fight against COVID-19.

## References

[CIT0001] British Medical Association. 2020a. Available from https://www.bma.org.uk/advice-and-support/covid-19/returning-to-the-nhs-or-starting-a-new-role/covid-19-staff-redeployment

[CIT0002] British Medical Association. 2020b. Available from https://www.bma.org.uk/advice-and-support/covid-19/ppe/covid-19-refusing-to-treat-where-ppe-is-inadequate

[CIT0003] British Medical Association. 2020c. Available from https://www.bma.org.uk/media/3070/bma-covid-tracker-survey-full-results-aug-2020.pdf

[CIT0004] British Orthopaedic Association. 2020. Available from https://www.boa.ac.uk/uploads/assets/ee39d8a8-9457-4533-9774e973c835246d/4e3170c2-d85f-4162-a32500f54b1e3b1f/COVID-19-BOASTs-Combined-FINAL.pdf

[CIT0005] Chang Liang Z , Wang W , Murphy D , Po Hui J H . Novel coronavirus and orthopaedic surgery: early experiences from Singapore. J Bone Joint Surg Am 2020; e000236. 10.2106/JBJS.20.00236 PMC7141583 32379113

[CIT0006] Culp B M , Frisch NB . COVID-19 impact on young arthroplasty surgeons. J Arthroplasty 2020; 35(7S): S42–S44. 10.1016/j.arth.2020.04.058 PMC7194608 32402577

[CIT0007] Gallagher T H , Schleyer A M . “We signed up for this!”—student and trainee responses to the Covid-19 pandemic. N Engl J Med 2020; 382(25): e96. 32268020 10.1056/NEJMp2005234

[CIT0008] Health Education England. 2020. Available from https://www.hee.nhs.uk/coronavirus-information-trainees

[CIT0009] ICNARC. 2020. Available from https://www.icnarc.org/Our-Audit/Audits/Cmp/Reports

[CIT0010] Public Health England. 2020. Available from https://www.gov.uk/government/publications/wuhan-novel-coronavirus-infection-prevention-and-control/covid-19-personal-protective-equipment-ppe#summary-of-ppe-recommendations-for-health-and-social-care-workers

[CIT0011] Sarpong N O , Forrester L A , Levine W N . What’s important: redeployment of the orthopaedic surgeon during the COVID-19 Pandemic: perspectives from the trenches. J Bone Joint Surg Am 2020; 102(12): 1019–21. 32287087 10.2106/JBJS.20.00574 PMC7224625

